# Comparison of Modifications in Flap Anastomosis Patterns and Skin Incision Types for External Dacryocystorhinostomy: Anterior-Only Flap Anastomosis with W Skin Incision versus Anterior and Posterior Flap Anastomosis with Linear Skin Incision

**DOI:** 10.1155/2015/170841

**Published:** 2015-06-21

**Authors:** Burcu Dirim, Selam Yekta Sendul, Mehmet Demir, Erdem Ergen, Zeynep Acar, Ali Olgun, Semra Tiryaki, Hakan Sensoz, Dilek Guven

**Affiliations:** Sisli Hamidiye Etfal Training and Research Hospital, Halaskargazi Avenue, Etfal Street, Sisli, 34371 Istanbul, Turkey

## Abstract

*Purpose*. To compare the outcomes of external dacryocystorhinostomy (E-DCR) by using two different flap anastomosis patterns and skin incision types. *Methods*. This study included 79 patients (88 eyes) with lacrimal drainage system disorders who underwent E-DCR surgery. Fifty eyes of 44 patients (group A) underwent E-DCR by suturing anterior and posterior flaps (H-flap) of the lacrimal sac with curvilinear skin incision whereas in 38 eyes of 35 patients (group B) DCR was performed by suturing only anterior flaps (U-flap) with W skin incision. *Results*. The success rate was evaluated according to lacrimal patency and scar assessment scores. Patency was achieved in 78 patients (88.6%). In terms of groups, patency was 44 eyes (88.0%) in group A and 34 eyes (89.5%) in group B. There was no statistically significant difference in the success rates of lacrimal patency between the two groups. Further, there was no statistically significant difference concerning cutaneous scar scores. *Conclusion*. Our study suggests that anastomoses of only anterior flaps or both anterior and posterior flaps have similar success rates; suturing only anterior flaps is easier to perform and shortens the operative time. In addition, W skin incision is a reasonable alternative to curvilinear incision for reducing scar formation.

## 1. Introduction

Primary acquired nasolacrimal duct obstruction is treated with dacryocystorhinostomy (DCR) surgery. The purpose of a DCR is to bypass the obstructed nasolacrimal system by creating an anastomosis between the lacrimal sac and the nasal mucosa. There are different methods for performing DCR including endoscopic and cutaneous approaches. One standard technique is known as external DCR (E-DCR) [1, 2]. Toti first described E-DCR in 1904 as an external approach to the sac through a skin incision in the medial canthus [3]. In 1921, Dupuy-Dutemps and Bourget and later Falk et al. in 1961 added lacrimal and nasal mucosal flap anastomosis as a modification to the original procedure [4, 5].

For the treatment of such patients alternative mucosal flap procedures have been performed [6, 7]. There are also a few studies comparing different skin incision types. In this study, we evaluated the outcomes of E-DCR by using two different techniques of flap anastomosis and medial canthal skin incision: one being the modified technique of only anterior flap anastomosis with W-shaped skin incision and the other being the conventional type of anterior and posterior flap anastomosis with curvilinear skin incision.

## 2. Materials and Methods

This study was a retrospective case series review of 79 patients (88 eyes) who underwent external DCR in our hospital between August 2010 and January 2014. Before surgery all patients underwent a routine ophthalmological examination and irrigation (syringing) to confirm obstruction of the lower lacrimal drainage system.

Patients with canalicular obstruction and previous lacrimal surgery were excluded. This study was conducted in accordance with the tenets of the Helsinki Declaration of 1975 (1983) and with the approval of an internal Institutional Ethical Committee.

Patients were then divided into two groups as follows: group A included 44 patients (50 eyes) who underwent DCR by both anterior and posterior flap (H-flap) anastomosis with curvilinear skin incision and group B included 35 patients (38 eyes) who underwent DCR by only anterior flap anastomosis (U-flap) with W skin incision.

In both groups an external DCR procedure was performed under general anesthesia. In the group A, a curvilinear incision 10–12 mm in length and, in the group B, a W-shaped incision 12 mm in length were made 6–8 mm medial to the medial canthus. Orbicularis muscle fibers were bluntly dissected to expose the periosteum overlying the anterior lacrimal crest. The medial canthal tendon and periosteum were incised and reflected with a periosteal elevator to expose the lacrimal fossa. An approximately square shaped 15 × 15 mm bony window was created with the use of a Kerrison punch. In group A, an H-shaped incision was made and both anterior and posterior flaps were closed using a 6.0 Vicryl (polyglactin) absorbable suture. In group B, only anterior flaps were sutured. In addition, scars, stenosis, or membrane on the distal common canaliculus was corrected by passing a silicone rod through the lacrimal drainage pathway. At the end of the procedure orbicularis muscle and skin were closed and 6.0 polyglactin suture was used for flap anastomosis and muscle layer whereas 6.0 polypropylene monofilament nonabsorbable suture was used for both types of skin incision.

Postoperatively, corticosteroid and antibiotic eye drops were administered 4 times daily for 10 days and oral antibiotics were administered twice daily for 1 week. Skin sutures were removed 10 days after surgery. Each patient was examined on the day of the operation for evidence of hemorrhage. Follow-up visits were scheduled in a period of 18 months from the date of the surgery.

Each visit included questioning the presence of epiphora and evaluation of the patency of lacrimal passage by syringing (irrigation). Surgical success was defined as patient satisfaction about the incision scar, relief of epiphora, and lacrimal patency.

Scar scoring was performed by observation with direct lighting with a 100 cm distance from the patient in the same room. The invisibility of the scar from a distance of 100 cm was considered as grade 1, minimal visibility grade 2, moderate visibility grade 3, and high visibility grade 4.

### 2.1. Statistical Analysis

Statistical Package for the Social Sciences version 22.0 software (SPSS Inc., Chicago, IL, USA) was used for statistical analysis. The distribution of the variables was evaluated by Kolmogorov-Smirnov test. In the analysis of quantitative and qualitative data, independent sample *t*-test, Mann-Whitney *U* test, and Chi-square test were used.

## 3. Results

The demographic data of the patients is given in Table 1.

In terms of gender, the majority of the treated patients, that is, 57 (64.8%), were female and 31 (35.2%) were male. In group A, 29 (58.0%) patients were female and 21 (42.0%) patients were male, while in group B, 28 (73.7%) patients were female and 10 (26.3%) patients were male. In group B, the number of the female patients was a bit higher than the number of the male patients but there was no significant difference between the two groups concerning sex (*p* = 0.127).

In terms of age, the mean ages were 53.3 ± 11.2 and 47.1 ± 17.8 in groups A and B, respectively, which showed no significant difference (*p* = 0.056).

The mean follow-up time for both groups was 13 months (range 6–18 months). The mean follow-up time for group A was 17.1 ± 12.4 months whereas it was 15.1 ± 9.2 months for group B. The difference was not statistically significant (*p* = 0.751).

Lacrimal system disorders in this study were chronic dacryocystitis and nasolacrimal duct obstruction in 78 (88.6%) cases, medial common canalicular obstruction in 7 (7.9%) cases, acute dacryocystitis in 2 (2.2%) cases, and lacrimal fistula in 1 (1.1%) case (Table 2).

Chronic dacryocystitis and nasolacrimal duct obstruction were the dominant indications in this research.

The success rate was evaluated according to lacrimal patency and scar assessment scores (Table 3).

Anatomic lacrimal patency was achieved in 78 patients (88.6%) whereas recurrence was observed in 10 cases (11.4%). With respect to groups, patency was 44 (88.0%) in group A and 34 (89.5%) in group B. Epiphora occurred in 6 cases (12.0%) in group A and in 4 cases (10.5%) in group B. The outcomes were statistically insignificant between the two groups (*p* = 0.829). Also there was no statistically significant difference between the two groups concerning the incision scar scores (*p* = 0.141).

## 4. Discussion

External dacryocystorhinostomy (E-DCR) is widely accepted standard surgical procedure for the treatment of primary acquired nasolacrimal duct obstruction [8, 9]. The reported success rate varies between 85% and 99% [10]. E-DCR is a reliable but difficult surgical technique and requires considerable experience. Also, incisional scar tissue after E-DCR may represent a cosmetic problem for patients.

In this study we compared two techniques in separate groups, one being the anastomosis of only anterior flaps with W skin incision and the other being suturing both anterior and posterior flaps with linear skin incision.

The success of DCR depends on the creation of a mucosa-lined anastomosis. Granulation tissue and fibrous scar formation is the natural inflammatory response of the body. It has been usually implied that suturing both anterior and posterior flaps reduces the mucosal scarring [11]. However, suturing posterior flaps often constitutes a difficulty and may take long operative times particularly in the presence of bleeding during surgery [12]. Although suturing only anterior flaps is easier this technique shows a success rate comparable to that obtained by suturing anterior and posterior flaps.

Baldeschi et al. [13] compared 3 different patterns of mucosal flap design (anterior and posterior, extended anterior with posterior, and only anterior) in external DCR. They found that the success rates among the 3 groups were similar.

Another study suggests that external DCR by suturing anterior and posterior flaps has no advantage over dacryocystorhinostomy by suturing only anterior flaps [14]. In addition, Haefliger et al. [15] suggest that the excision of nasal and lacrimal sac mucosae in front of the osteotomy of an external DCR does not associate with a large number of postoperative surgical failures. Türkcü et al. found that anastomosis of only posterior flaps does not affect the success rate of external DCR and is technically simpler [16]. Serin et al. reported that with posterior flap anastomosis success rate was 93.75% and with resection it was 96.67% [17]. Khan et al. reported a success rate of 97.1% in DCR by suturing the posterior flaps and 94.3% in DCR by excision of the posterior flaps [18]. In our study, in group B, we preferred the excision of posterior flaps instead of leaving them like Serin and Khan et al. did.

Scar formation is a frequently cited complication of E-DCR. Curvilinear incision avoids scar formation as much as possible and follows the relaxed skin tension lines better and with less webbing in E-DCR [19]. Also W incision may be considered as a modified form of Z-plasty and Ekinci et al. showed that W incision is effective in reducing incisional scarring by relaxing skin tension in patients undergoing E-DCR [20]. In our study mean assessment scores for both curvilinear and W incisions were similar in two groups.

In conclusion, our study suggests that anastomoses of only posterior flaps or both anterior and posterior flaps have similar success rates that suturing only anterior flaps and excision of posterior flaps are easier to perform and shorten the operative time. Also, W incision may be a good alternative to curvilinear incision for reducing scar formation after E-DCR.

## Figures and Tables

**Table 1:** Demographic features of groups A and B.

	Group A (*n* = 50)	Group B (*n* = 38)	*p* value
Age (years) (mean ± sd)	53.3 ± 11.2	47.1 ± 17.8	0.056
Gender			
Male (%)	21 (42.0%)	10 (26.3%)	0.127
Female (%)	29 (58.0%)	28 (73.7%)	

**Table 2:** Lacrimal drainage system disorders by groups.

Lacrimal drainage system disorders	Group A	Group B	Total (%)
Chronic dacryocystitis and nasolacrimal duct obstruction	45	33	78 (88.6)
Acute dacryocystitis	1	1	2 (2.2)
Medial common canalicular obstruction	3	4	7 (7)
Lacrimal fistula	1	0	1 (1.1)

**Table 3:** The mean assessment of scar scores for both groups.

Mean scar scores	Group A (%)	Group B (%)
Grade 1	30 (60.0%)	19 (50.0%)
Grade 2	8 (16.0%)	14 (36.8%)
Grade 3	5 (10.0%)	2 (5.3%)
Grade 4	7 (14.0%)	3 (7.9%)

## References

[1] Yazici B., Akova B. (2007). Simultaneous bilateral external dacryocystorhinostomy. *Acta Ophthalmologica Scandinavica*.

[2] Boboridis K. G., Bunce C., Rose G. E. (2005). Outcome of external dacryocystorhinostomy combined with membranectomy of a distal canalicular obstruction. *American Journal of Ophthalmology*.

[3] Tarbet K. J., Custer P. L. (1995). External dacryocystorhinostomy. Surgical success, patient satisfaction, and economic cost. *Ophthalmology*.

[4] Hughes S. M. (1986). The history of lacrimal surgery. *Advances in Ophthalmic Plastic & Reconstructive Surgery*.

[5] Dupuy-Dutemp L., Bourget M. (1921). Note preliminaire sur en procede de dacryocystorhinostomie. *Annales d'Oculistique*.

[6] Baldeschi L., MacAndie K., Hintschich C. R. (2004). The length of unsutured mucosal margins in external dacryocystorhinostomy. *The American Journal of Ophthalmology*.

[7] Erdoğan G., Ünlü C., Turan Vural E., Aykut A., Bayramlar H. (2010). Inferior flap anastomosis in external dacryocystorhinostomy. *Ophthalmic Plastic and Reconstructive Surgery*.

[8] Tarbet K. J., Custer P. L. (1995). External dacryocystorhinostomy: surgical success, patient satisfaction, and economic cost. *Ophthalmology*.

[9] Welham R. A. N., Henderson P. H. (1973). Results of dacryocystorhinostomy: analysis of causes for failure. *Transactions of the Ophthalmological Societies of the United Kingdom*.

[10] Cokkeser Y., Evereklioğlu C., Er H. (2000). Comparative external versus endoscopic dacryocystorhinostomy: results in 115 patients (130 eyes). *Otolaryngology—Head and Neck Surgery*.

[11] Serin D., Alagöz G., Karslıoğlu Ş., Çelebi S., Kükner Ş. (2007). External dacryocystorhinostomy: double-flap anastomosis or excision of the posterior flaps?. *Ophthalmic Plastic and Reconstructive Surgery*.

[12] Erdoğan G., Ünlü C., Vural E. T., Aykut A., Bayramlar H. (2010). Inferior flap anastomosis in external dacryocystorhinostomy. *Ophthalmic Plastic and Reconstructive Surgery*.

[13] Baldeschi L., MacAndie K., Hintschich C. R. (2004). The length of unsutured mucosal margins in external dacryocystorhinostomy. *American Journal of Ophthalmology*.

[14] Kaçaniku G., Begolli I. (2014). External dacryocystorhinostomy with and without suturing the posterior mucosal flaps. *Medicinski Arhiv*.

[15] Haefliger I. O., Tschopp M., Pimentel A.-R. (2012). Mucosal excision instead of fashioning nasolacrimal mucosae flaps during external dacryocystorhinostomy: a pilot study. *Klinische Monatsblätter für Augenheilkunde*.

[16] Türkcü F. M., Öner V., Taş M., Alakuş F., Işcan Y. (2012). Anastomosis of both posterior and anterior flaps or only anterior flaps in external dacryocystorhinostomy. *Orbit*.

[17] Serin D., Alagöz G., Karsloğlu Ş., Çelebi S., Kükner Ş. (2007). External dacryocystorhinostomy: Double-flap anastomosis or excision of the posterior flaps?. *Ophthalmic Plastic and Reconstructive Surgery*.

[18] Khan F. A., Yaqub M. A., Fayyaz M. (2010). The importance of excising or suturing the posterior mucosal flaps in external dacryocystorhinostomy. *Pakistan Journal of Ophthalmology*.

[19] Dave T. V., Ali M. J., Sravani P., Naik M. N. (2012). Subciliary incision for external dacryocystorhinostomy. *Ophthalmic Plastic and Reconstructive Surgery*.

[20] Ekinci M., Cagatay H. H., Gokce G. (2014). Comparison of the effect of W-shaped and linear skin incisions on scar visibility in bilateral external dacryocystorhinostomy. *Clinical Ophthalmology*.

